# Bio-inspired synthesis of aqueous nanoapatite liquid crystals

**DOI:** 10.1038/s41598-018-36843-w

**Published:** 2019-01-24

**Authors:** Junjun Tan, Xiaoying Jin, Minfang Chen

**Affiliations:** 1 Hubei Province Key Laboratory of Green Materials for Light Industry, Collaborative Innovation Center for Green Light-weight Materials and Processing, Hubei University of Technology, Wuhan, 430068 P. R. China; 2 School of Materials and Chemical Engineering, Hubei University of Technology, Wuhan, 430068 Hubei P. R. China; 3 School of Materials Science and Engineering, Tianjin University of Technology, Tianjin, 300384 P. R. China

## Abstract

The macroscopically ordered structure of rod-like nanoapatites within the collagen matrix is of great significance for the mechanical performance of bones and teeth. However, the synthesis of macroscopically ordered nanoapatite remains a challenge. Inspired by the effect of citrate molecules on apatite crystals in natural bone and the similarities between these ordered rod-like nanoapatites and the nematic phase of inorganic liquid crystals (LCs), we synthesized aqueous liquid crystal from rod-like nanoapatites with the aid of sodium citrate. Following a similar procedure, aqueous Mg(OH)_2_ and Mg_3_(PO_4_)_2_ LCs were also prepared. These findings lay the foundation for the fabrication of macroscopically assembled nanoapatite-based functional materials for biomedical applications and offer a green chemical synthesis platform for the development of new types of inorganic LCs. This process may reduce the difficulties in synthesizing large quantities of inorganic LCs so that they can be applied to the fabrication of functional materials.

## Introduction

In materials science, the ordering of a single specimen in a continuous phase on the nanometer scale is of particular interest because of the outstanding optical and mechanical properties of such hybrids or nanocomposites. Bone tissues are a peculiar natural example of an ordered nanocomposite. They possess highly organized hierarchical structures in which the basic building blocks (apatite crystals) are generally in the nanometer size range to ensure optimal physical and biological function^1^. Considerable research has been conducted on the preparation of apatite nanocomposites that mimic the natural properties of bone, so that they can be used for bone repair or fixation^2–4^. However, the preparation of macroscopically ordered materials through the self-organization or self-assembly of rod-like nanoapatite in polymer or protein matrixes remains a challenge due to the lack of a scalable assembly method.

Since Zocher’s pioneering work on the preparation of V_2_O_5_ nanobelts liquid crystals (LCs)^5,6^, it has been recognized that anisotropic colloidal particles in a dispersion may undergo a phase transition from the isotropic state to the liquid crystalline state if the particle concentration exceeds a critical value. This shows great similarity to natural bone tissue structure. Thus, if nanoapatite LCs can be synthesized on a large scale, the problem mention above could be solved, and apatite-based nanocomposites would give full play to desired mechanical performance. To the best of our knowledge, the assembly of nanoapatite LCs has not previously been reported.

Onsager^7^ first provided an explanation for the formation of colloidal plate and rod LCs in dispersions: the loss of orientational entropy associated with particle alignment is overcome by the simultaneous gain in excluded volume (configurational) entropy. Generally, an appropriate aspect ratio (length/diameter ratio), sufficient colloidal stability, and a critical particle concentration are three basic terms which should be met to allow the formation of LCs. A few colloidal liquid crystals have been successfully prepared under the guidance of Onsager’s theory, including gibbsite nanoplates^8^, carbon nanotubes^9^, oxide graphene^10,11^, TiO_2_ nanorods^12^, LDH^13^, SiO_2_ nanorods^14^, and others.

Solid state NMR analysis of natural bone has shown an abundance of citrate molecules in bone tissue—about 5.5 wt% of the organic matter in bone—and most of these molecules are on the apatite surface, with a density of about one molecule per two square nanometers of apatite crystalline surface^15^. These citrate molecules play very important roles in facilitating the dispersion of rod-like nanoapatites in bone tissue and in controlling the growth of apatite crystals. These functions of the citrate molecules meet the requirements for LC formation perfectly. In fact, sodium citrate is commonly used as an anti-thickening and dispersing agent in typical artificial chemical syntheses^16^.

Considerable effort has been put forth to investigate the various factors that affect the formation of HA in the citrate-assisted hydrothermal method. These factors include the molar ratio of calcium to citrate^17,18^, hydrothermal temperature^19^, hydrothermal time^19^, synergistic effect of citrate with other compounds^20–22^, and ion doping^23–25^. By adjusting these factors, the morphology, size, crystallinity, and colloidal stability of the resultant nanocrystalline apatite can be greatly controlled^19^. Owing these merits, these material has been successfully applied in the drug delivery and show excellent bio-safety^26–28^. Based on the previous literature and our previous findings, it seems reasonable to infer that the formation of liquid crystalline nanoapatite in water should be achievable through chemical synthesis with the aid of citrate molecules.

Herein, we report the bio-inspired synthesis of nanoapatite LCs based on the assembly of water-dispersible rod-like nanoapatite. The method allows precise control of the sizes, aspect ratios, and surface properties of the nanoapatite LCs by adjusting the mole ratio of sodium citrate to calcium salts (R_C/C_), the hydrothermal temperature, and the hydrothermal time. Moreover, in consideration of the strong complex-forming interactions between citrate and many metal ions, this strategy can be easily applied to other, as yet unreported LC systems.

## Materials and Methods

### Materials

Calcium nitrate tetrahydrate (≥99.0%), magnesium nitrate hexahydrate (≥99.0%), sodium phosphate tribasic dodecahydrate (≥98.0%), sodium citrate tribasic dihydrate (≥99.0%), ethanol absolute (≥99.7%) and NaOH (≥96.0%) were purchased from Sinopharm Chemcial Reagent Co. Ltd (Shanghai, China). All chemicals were used as received without further purification. Deionized water was used throughout.

### Preparation of colloidal nanoapatites dispersions

Colloidal nanoapatites were prepared using a hydrothermal method. In a typical experiment, a C_6_H_5_Na_3_O_7_·2H_2_O solution of fixed concentration (0–0.0067 M, 10 mL) was slowly added to an aqueous solution of Ca(NO_3_)_2_·4H_2_O (0.01 M, 15 mL) with continuous stirring over 10 min. Then, an aqueous solution of Na_3_PO_4_·12H_2_O (0.006 M, 15 mL) was progressively added to the mixture with vigorous stirring over 15 min. After that, the mixed solution was transferred, as obtained, to a Teflon-lined stainless steel autoclave with a 50 mL capacity. The solution in the autoclave underwent hydrothermal treatment at 90–180 °C for 3–24 h. After the hydrothermal treatment, the autoclave was allowed to cool down naturally and the resulting product was purified using a three-cycle centrifugation-washing (3700 *g*) process with deionized water and ethanol. Finally, the purified product was redispersed in deionized water to form an aqueous dispersion. The pH was adjusted to pH 9 by the addition of 0.1 M NaOH. The dispersion was concentrated with a rotary evaporator and then diluted into a series of samples with different particle concentrations. These dispersions were judged as follows: a 1-mL sample of a typical dispersion was taken using a pipette, and then the pipette tip was dried in a vacuum oven at 60 °C for 24 h. The particle concentrations of hydroxyapatite nanorods in the dispersions were calculated using the differences between the weights of the original pipette tips and those of the tips that were dried after adding the dispersion.

Mg(OH)_2_ and Mg_3_(PO_4_)_2_ nanoplates were synthesized using a procedure similar to the one described above for hydroxyapatite nanorods. For the preparation of Mg(OH)2 nanoplates, we replaced the Ca(NO_3_)_2_·4H_2_O and Na_3_PO_4_·12H_2_O solutions with Mg(NO_3_)_2_·4H_2_O (0.01 M, 15 mL) and NaOH (0.02 M, 15 mL) solutions. For the preparation of Mg_3_(PO_4_)_2_ nanoplates, we replace the Ca(NO_3_)_2_·4H_2_O solution with a Mg(NO_3_)_2_·4H_2_O solution (0.01 M, 15 mL). In each case C_6_H_5_Na_3_O_7_·2H_2_O (0.0067 M, 10 mL) was used. The hydrothermal temperature and hydrothermal time were 150 °C and 3 h, respectively. The follow-up process was the same.

### Characterization of hydroxyapatite liquid crystal

#### Wide-angle X-ray diffraction (XRD)

The dried powders were characterized using X-ray diffraction with an X’Pert PRO X-ray diffractometer (PANalytical B.V., Almelo, Netherlands). Analyses were performed using a Cu Kα radiation (1.5406 A) source at 60 kV and 60 mA from 5° to 80° with a scan rate of 0.5°/min.

#### Transmission Electron Microscopy (TEM)

The morphology of each product was inspected using a Tecnai G2/F20 Transmission Electron Microscope (TEM) (FEI, Hillsboro, OR, USA) with an accelerating voltage of 200 kV. Each colloidal hydroxyapatite nanoparticle samples was drawn by pipette and 1–2 droplets were placed onto the carbon side of a holey carbon-coated Cu TEM-grid. The mean length (L) and diameter (D) of the hydroxyapatite nanorods were measured using a statistical method from three random TEM images (at least 300 counts). Therefore, it is possible to define the R_L/D_ using the equation:

$${{\rm{R}}}_{{\rm{L}}+{\rm{D}}}=\frac{\sum _{1}^{{\rm{n}}}{{\rm{L}}}_{{\rm{i}}}/{{\rm{D}}}_{{\rm{i}}}}{{\rm{n}}}({\rm{n}} > 300)$$

where the parameters L_i_ and D_i_ were defined as the length and the diameter of each nanoapatites respectively. The R_L/D_ is used to evaluate the aspect ratio of hydroxyapatite nanorods.

#### Inductively Coupled Plasma Optical Emission Spectrometry (ICP-OES)

The elemental contents of Ca and P in the samples were determined by Inductively Coupled Plasma Optical Emission Spectrometry (ICP-OES, Agilent 725, Agilent Technologies Co. Ltd., USA).

#### Thermogravimetric analysis (TGA)

The measurement was carried out using a Thermal Analysis SDT Q 600 (TA Instruments, New Castle, DE, USA). Heating was performed in a nitrogen flow (100 ml min^−1^) using an alumina sample holder. Temperature was increased from room temperature to 1200 °C with a heating rate of 10 °C min^−1^. Sample weight was about 5 mg.

#### Zeta Potential and DLS Measurements

Zeta potential and particle size distribution measurements were performed using a nano-ZS90 Zetasizer (Malvern instruments Ltd., Malvern, UK). Particle size distributions were measured after equilibration for 2 min at 25 °C using disposable polystyrene cuvettes containing dispersions (0.01 g/mL). The data were recorded and analyzed using the Dispersion Technology Software v. 5.0 (Malvern Instruments). For zeta potential measurement, samples (0.5 wt% particle dispersion) were deposited into clear disposable zeta cells and the results were automatically generated with the help of Dispersion Technology Software v. 5.0 (Malvern Instruments). The sample pH, which was modified using NaOH or HCl (0.1 M), was determined using a PB-10 pH meter (Sartorius AG, Goettingen, Germany) at 25 °C. No additional electrolytes were added to each sample for the measurement of zeta potential and particle size distribution.

#### Birefringence observation

The macroscopic birefringence of nanoapatite dispersions with different particle concentrations were studied between crossed polarizers in sealed cuvettes with a width of 1 mm that were filled with about 0.2 cm^3^ of dispersion. The microscopic birefringence of the hydroxyapatite dispersions with different particle concentrations were examined using an XP-213 polarizing microscope (Nanjing Jiangnan Novel Optics Co., China). Samples were sealed between a hollow glass slide and cover slip to prevent solvent evaporation (sample thickness of about 100 μm).

## Results and Discussions

**Figure 1:**
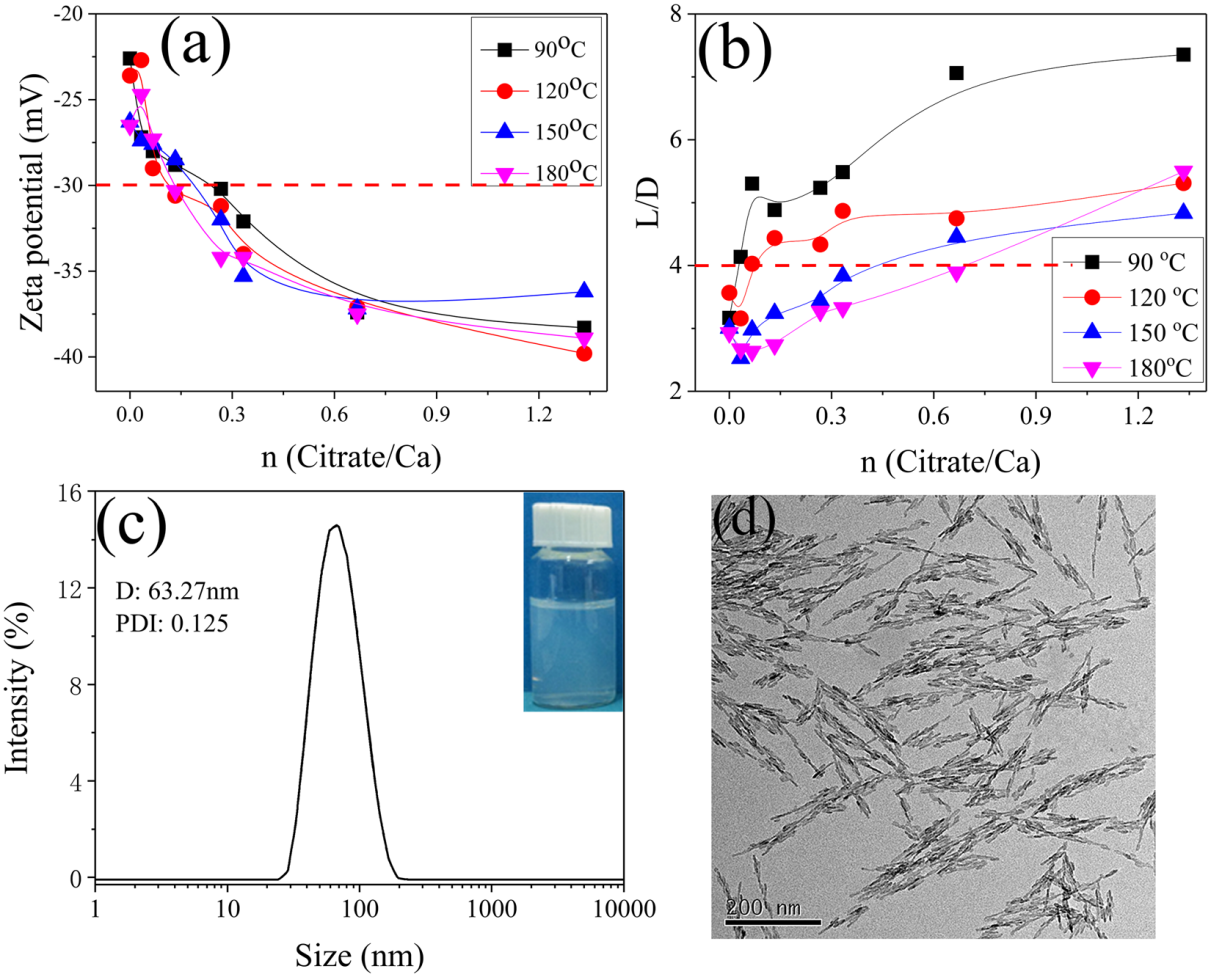
Effects of the citrate to calcium ratio (R_C/C_) and the hydrothermal temperature on (**a**) Zeta potential and (**b**) statistical R_L/D_ of rod-like nanoapatite synthesized by hydrothermal method with the aid of sodium citrate at different experimental conditions. (**c**) Representative dynamic light scattering and (**d**) transmission electron microscopic images of synthesized nanoapatite at hydrothermal temperature 150 °C, hydrothermal time 3 h and R_C/C_ 2/3(inner picture of (**c**) is nanoapatite dispersion with particle concentration of 2 wt%).

To access aqueous nanoapatite LCs, the most vital step is to prepare nanoparticles with a high length to diameter ratio (R_L/D_) and sufficient colloidal stability in water. Therefore, we systematically evaluated the zeta potentials and statistical R_L/D_s of nanoapatites synthesized at different R_C/C_s and hydrothermal temperatures (Fig. 1a,b). The absolute values of the zeta potential and R_L/D_ increased with increasing R_C/C_, while the absolute value of the zeta potential showed no obvious change and the R_L/D_ decreased dramatically with increasing hydrothermal temperature (from 90 to 180 °C). Considering the colloidal stability of the nanoapatite dispersion and the aspect ratio of the rod-like nanoapatites, an R_C/C_ above 2/3, a hydrothermal time of 3 h, and a hydrothermal temperature above 90 °C should be appropriate for the preparation of liquid crystalline nanoapatite (for detailed morphology evolution, see Fig. S1). Under these conditions, the R_L/D_ reaches 7 and the absolute values of zeta potential reaches −35 mV.

To further optimize experimental procedure, a hydrothermal temperature of 150 °C, an R_C/C_ of 2/3, and an experimental time 3 h were chosen as typical experimental condition for the preparation of nanoapatite LCs. This optimization was guided by the similar effects of increasing hydrothermal temperature and prolonging hydrothermal time on nanoapatite crystal growth^19^. The morphology of the nanoapatites produced under these experimental conditions was characterized using dynamic light scattering and transmission electron microscopy (Fig. 1c,d). These rod-like nanoapatites have an average length of 11.9 nm and an average lateral diameter (D) of 60.6 nm, with a corresponding R_L/D_ of 5.3. The crystalline structure of the sample obtained was characterized by XRD (Fig. S2). All of the diffraction peaks in the XRD pattern can be easily indexed to a pure hexagonal-phase hydroxyapatite, which is in good agreement with the reported data (JCPDS files, PDF No. 86-740) and the literature^29^. The Ca/P ratio of the sample was measured by ICP-OES and its value was 1.56, which was slightly lower than the stoichiometric value (1.67). Therefore, this method yields Ca-deficient apatite with a Ca/P ratio close to the biological ones. The content of citrate and carbonate in the sample was detected by thermogravimetric analysis. The weight losses curve can be mainly divided into three stages (Fig. S3): (i) from room temperature to 305 °C due to the adsorbed water and structural water, (ii) from 305 °C to 780 °C related to the citrate as well as the small amount of non-apatitic HPO4 ions, (iii) from 780 °C to 1000 °C corresponding to the carbonate ions^30^. According to thermogravimetric data, the sample contains 8.01 wt. % citrate and 1.65 wt. % carbonate. When these rod-like nanoapatites were dispersed into an aqueous phase, the zeta potential value was −34 mV and hydrodynamic diameter was 63 nm, with a polydispersity index (PDI) of 0.125. This type of dispersion appears transparent under light blue and is colloidally stable for as much as a few months. rod-like nanoapatites of high R_L/D_ and excellent colloidal stability, like these, should meet the requirements to form LCs and lay the foundation for detailed studies of their possible liquid crystalline behavior.

Generally, the evolution of birefringence between polarizers upon concentration of the dispersion can be used as direct evidence of the formation of lyotropic LCs^6,30,31^. The birefringence of hydroxyapapatite dispersions with different particle concentrations was studied between crossed polarizers in cuvettes with a width of 1 mm containing approximately 0.2 cm^3^ of dispersion.

**Figure 2:**
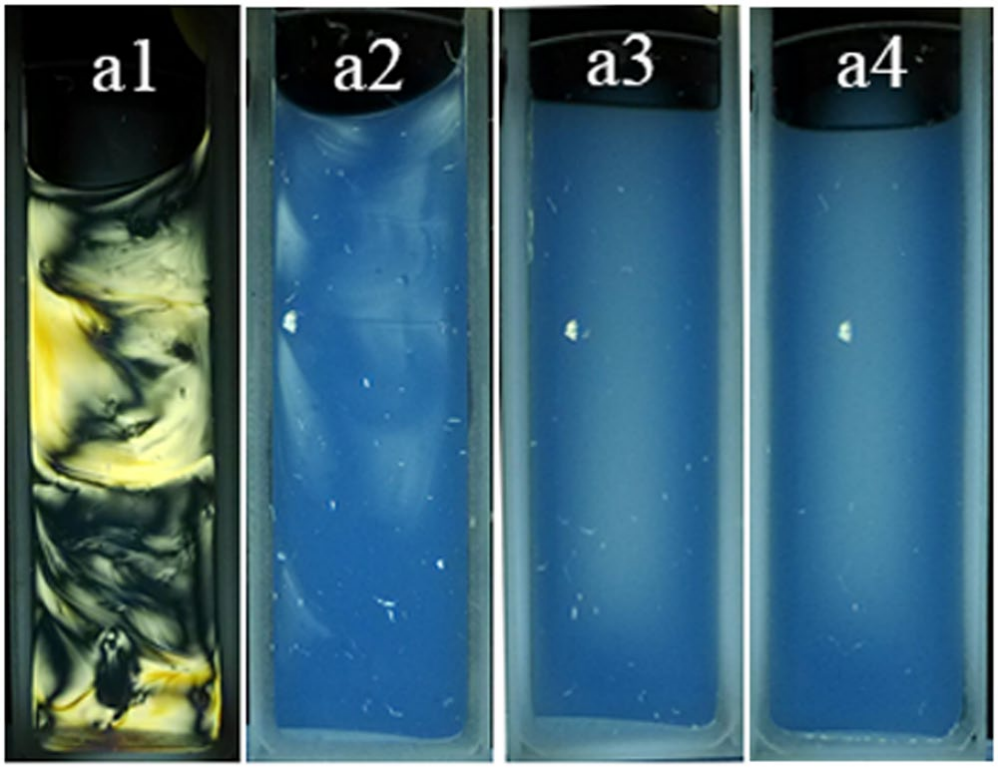
Macroscopic photographs (**a**) between crossed polarizers of nanoapatite aqueous dispersions in cuvettes with particle concentration (g/mL) of (**a1**) 30.3%, (**a2**) 24.3%, (**a3**) 19.4%, (**a4**) 15.5% respectively.

When the particle concentration of the dispersion was below 19.4%, an isotropic phase was observed between the crossed polarizers (Fig. 2a). Permanent birefringence domains emerged when the particle concentration was increased to 24.2%. With a further increase in the dispersion concentration to 30.3%, stable birefringence spread to the entire dispersion, which displayed a vivid Schlieren texture typical of nematic phases. Moreover, at high particle concentrations like this, the dispersion rapidly turned into gelatin and no phase separation was observed. In addition to these macroscopic observations, the textures of the liquid-crystalline phases were also observed using a polarization microscope (Fig. S4).

**Figure 3:**
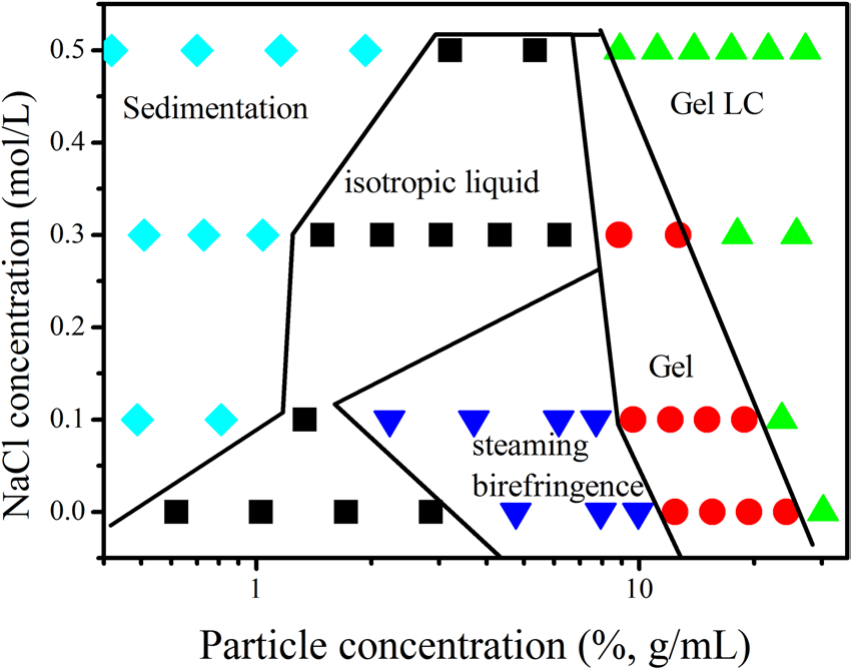
Phase diagram of aqueous nanoapatite dispersions with varying particle concentration and NaCl concentrations.

To fully understand the phase behavior of these nanoapatite dispersions, we established the phase diagram as a function of the rod-like nanoapatite and NaCl concentrations (Figs 3 and S5).

In the absence of NaCl, the nanoapatite dispersions evolve into four phases: an isotropic phase (particle concentration < 2.9%), a steaming birefringence phase (4.8% < particle concentration < 9.9%), a gel phase (12.4% < particle concentration < 24.3%) and a gel LC phase (particle concentration > 24.3%). Upon addition of 0.1 M NaCl to particle dispersions with a particle concentration < 0.81%, a new phase called the sedimentation phase, appears. When the NaCl concentration is increased to 0.3 M, the steaming birefringence phase disappears. When the NaCl concentration is increased to 0.5 M, the gelatin area disappears. It is worth noting that along with the increase in NaCl concentration, the particle concentration range of the LC expands gradually and the critical particle concentration of the phase transition moves to a lower particle concentration. From the point of view of the dominant interaction between particles, increasing salt concentration weakens the repulsive forces and induces phase evolution. This explanation can be confirmed by tracking the zeta potential and dynamic light scattering of the dispersions with increasing NaCl concentration (Fig. S4).

As previously discussed, nanoapatite LCs could be successfully prepared because of the formation of strong complexes between citrate molecules and calcium ions, which makes it easy to meet the requirements for the formation of LCs. Because it is well known that the strong interactions enable citrate ions to form complexes with many different metal ions, this case is more than an exception. The procedure should allow the design of many new types of LCs. As examples, LCs were successfully synthesized from Mg(OH)_2_ and Mg_3_(PO_4_)_2_ nanoplates (Figs S5 and S6).

## Conclusions

In summary, inspired by the biological structure of bone, nanoapatite LCs has been successfully prepared with the aid of sodium citrate. The synthetic procedure is simple, scalable, cost effective and green. To the best of our knowledge, this report provides the first description of the synthesis of nanoapatite LCs. In addition, based on the universality of the interaction between metal ions and citrate molecules, this synthetic procedure could be applied to the preparation of other unreported inorganic LCs, such as Mg(OH)_2_ and Mg_3_(PO_4_)_2_ nanoplates. These novel findings not only lay a foundation for the fabrication of macroscopically assembled nanoapatite-based functional materials for biomedical applications, they also offer a green chemical synthesis platform to develop new types of inorganic LCs. This may reduce the difficulty in synthesizing large quantities of inorganic LCs so that inorganic LCs can be applied more easily to the fabrication of functional materials.

## Electronic supplementary material

Supporting information
