# Supplementary material for: Bio-inspired synthesis of aqueous nanoapatite liquid crystals
Source: Sci Rep. 2019 Jan 24;9:466. doi: 10.1038/s41598-018-36843-w (PMC6345739; doi:10.1038/s41598-018-36843-w)
Supplement: Supplementary file 1 — Supporting information [file 41598_2018_36843_MOESM1_ESM.docx]

Supporting information

**Bio-inspired synthesis of aqueous nanoapatite liquid crystals**

Junjun Tan ^ab^*, Xiaoying Jin ^b^, Minfang Chen ^c^*

**Corresponding author (*****):**

Junjun Tan;

Minfang Chen;

**Affiliations:**

a. Hubei Province Key Laboratory of Green Materials for Light Industry, Collaborative Innovation Center for Green Light-weight Materials and Processing, Hubei University of Technology, Wuhan 430068, P. R. China

b. School of Materials and Chemical Engineering, Hubei University of Technology, Wuhan, 430068, Hubei, P. R. China

c. School of Materials Science and Engineering, Tianjin University of Technology, Tianjin, 300384, P. R. China

**Tel:** 0086 27 59750460

**Fax:** 0086 27 59750482

**Corresponding author e-mail address:** [tanjunjun2011@hbut.edu.cn](mailto:tanjunjun2011@hbut.edu.cn)

[mfchentj@126.com](mailto:mfchentj@126.com)





































































Figure S1: TEM images of nanoapatites synthesized with different R_CC_ values (1-8 represent 0, 1/30, 1/15, 2/15, 4/15, 1/3, 2/3, and 4/3, respectively) at different temperatures (a-d represent 90 °C, 120 °C, 150 °C, 180 °C) with a hydrothermal time of 24 h.

Figure S2: XRD patterns of nanoapatites with an R_CC_ of 2/3, synthesized at 150 °C for 3 h.

Figure S3: TG curve of nanoapatites synthesized with an R_CC_ of 2/3 at 150 °C for 3 h.


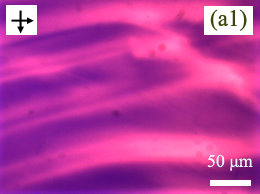

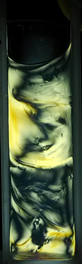

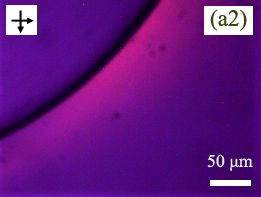

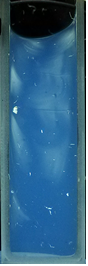


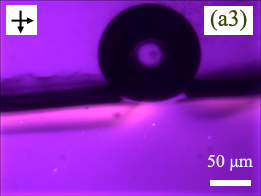

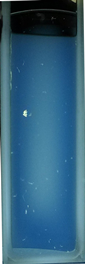

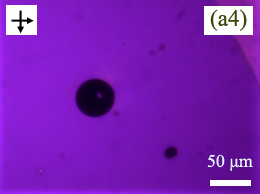

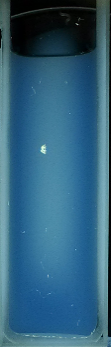


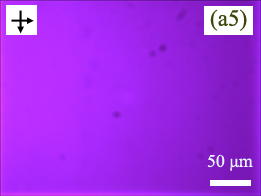

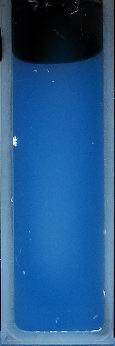

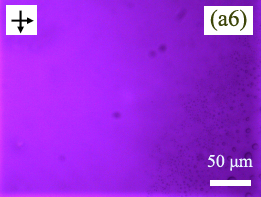

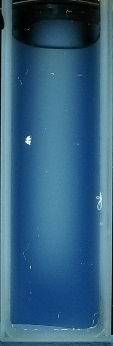


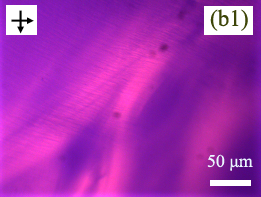

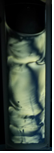

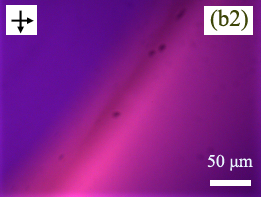

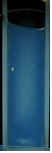


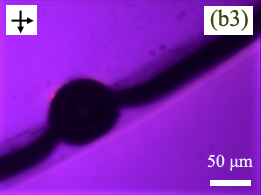

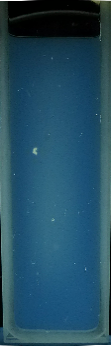

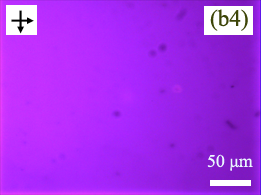

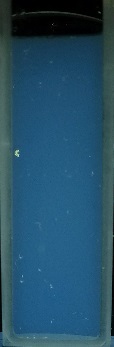


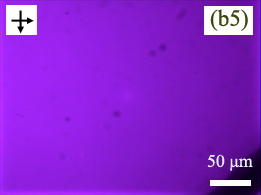

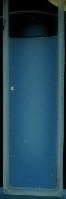

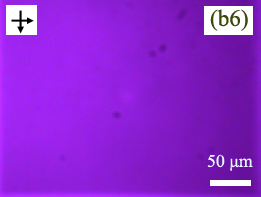

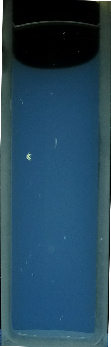


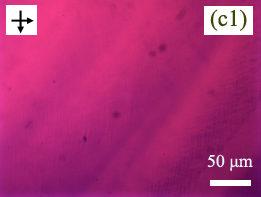

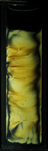

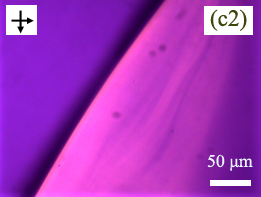

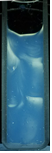


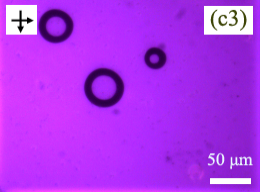

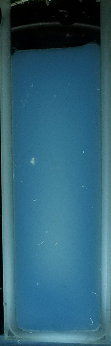

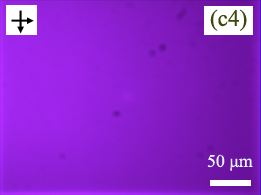

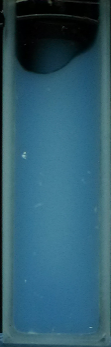


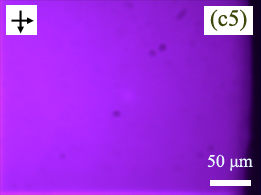

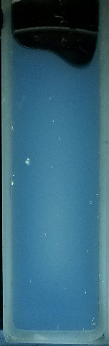

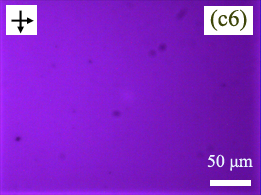

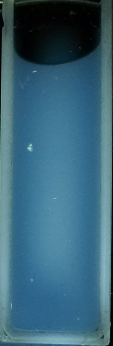


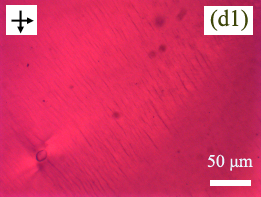

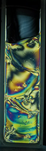

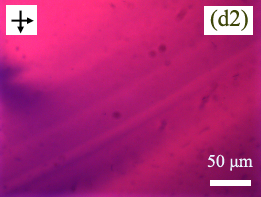

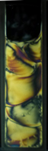


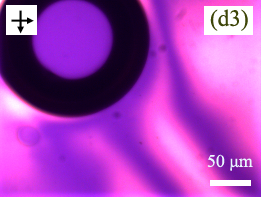

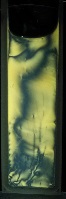

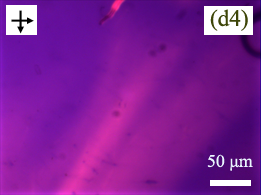

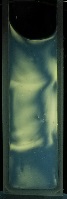


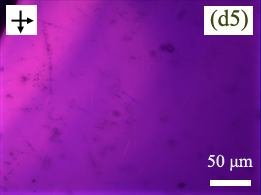

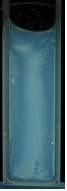

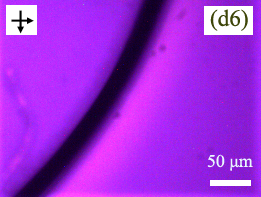

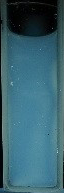


Figure S4: Polarized optical microscopic and macroscopic images of nanoapatite aqueous dispersions with different salt concentrations and different particle concentrations. (a) Salt-free, with particle concentrations (a1 to a6) of 30.3%, 24.3%, 19.4%, 15.5%, 12.4%, and 9.9% (g/cm^3^), respectively; (b) 0.1M NaCl with particle concentrations (b1 to b6) of of 23.6%, 18.9%, 15.1%, 12.1wt%, 9.7%, and 7.7% (g/cm^3^), respectively; (c) 0.3M NaCl with particle concentrations (c1 to c6) of 25.8%, 18.1%, 12.7%, 8.9%, 6.2%, and 4.4% (g/cm^3^), respectively; (d) 0.5M NaCl with particle concentrations (d1 to d6) of 27.2%, 21.8%, 17.4%, 13.9%, 11.2%, and 8.9% (g/cm^3^), respectively.

Figure S5: (a) Zeta potential and (b) dynamic light scattering of nanoapatite dispersions (0.5 wt%, pH 9) at different NaCl concentrations.

Figure S6: XRD patterns of (a) Mg(OH)_2_ and (b) Mg_3_(PO_4_)_2_ synthesized at 150 °C for 3 hours.


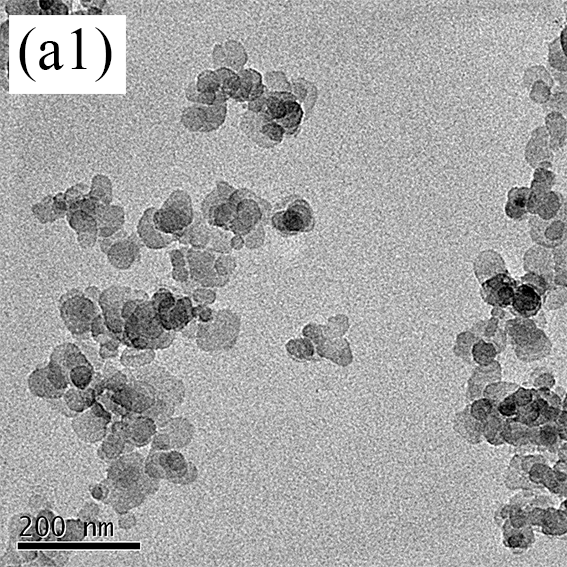

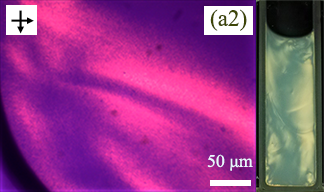


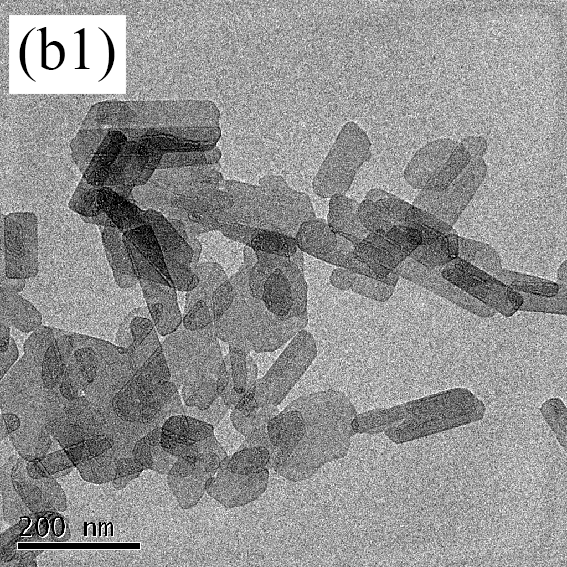

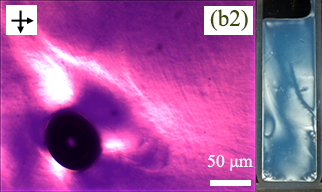


Figure S7: TEM images of Mg(OH)_2_ (a1) and Mg_3_(PO_4_)_2_ (b1) nanoparticles; Polarized optical microscopic and macroscopic images of Mg(OH)_2_ and Mg_3_(PO_4_)_2_ aqueous dispersions at particle concentrations of 15.6% (g/cm^3^) (a2) and 13.2% (g/cm^3^) (b2) respectively.
